# The Sunday Fund Meeting

**Published:** 1892-12-03

**Authors:** 


### Passing Topics.

### THE SUNDAY FUND MEETING.

It would be an entirely unreasonable proceeding for even the most ardent friend of Hospital Sunday to take umbrage at the suggestion that its success is not as full and conspicuous as it ought to be. For our own part we have no feeling but one of unalloyed satisfaction that by means of Hospital Sunday our religious bodies are afforded an opportunity of testifying to their participation in the practical benevolence which received its best and highest illustration in the Divine life on earth, while, on the other hand, our hospitals are brought by the same agency into close contact with those who are their natural allies. The principle comprehended in Hospital Sunday appears to our mind absolutely unassailable. And yet with all the wish in the world to think differently, we must continue to regard the Fund when tested by results, as illustrative of the indifference rather than the sympathy of the large majority of the congregations.

No charge of want of energy at headquarters, so far as we know, is ever hinted at. None the more do the efforts of the authorities appear capable of raising the total collection beyond the altogether inadequate sum at which it stands for the current year, the same, approximately, as it has been for some years past. A daily contemporary, in search for an explanation, appears to think that the demand of the hospitals is one too many for the householder already overburdened and weary of importunity. But while we are quite ready to admit that the claims in respect of citizenship are numerous and heavy, we altogether refuse to believe that an extra shilling placed once a year in the collection plate is likely to precipitate a financial catastrophe in an ordinary middle-class home, and we take leave to assert that a largely preponderating number of church and chapel goers are not one whit more inclined than other people to recognize their duty to our hospitals, without judicious prompting. If the negligent really do believe that they have a surfeit of benevolence, and that they would rather pay a hospital rate than contribute voluntarily, we think they would change their views as soon as the rate was levied. Neither do we assent to the suggestion that the hospitals are unpopular, because the very best evidence to the contrary is supplied in the ever increasing use of them. No, it is the old, old story; the inability of people to look at things from any point of view but their own—the poverty of the higher imaginative faculty.

Thus we reach the conclusion we desire to impress, by no means for the first time, upon the clergy whom it chiefly concerns. As soon as the preachers arouse in themselves a sense of all that hospitals are, and of all the public owe to them, and set themselves to place hospital work and hospital need before their congregations with graphic earnestness, then and not before, Hospital Sunday will be made a success. There is at once encouragement and reproof in the results achieved here and there by means such as we advocate. The clergy may not all be gifted alike, nor are the fields they cultivate equally fertile, but every minister can at least learn to put his heart into his appeal and to the utmost of his ability testify to the value of the cause he pleads for, and which he has so abundant opportunities to measure. Agricola

